# Unhealthy food and beverage marketing to children on digital platforms in Aotearoa, New Zealand

**DOI:** 10.1186/s12889-022-14790-6

**Published:** 2022-12-22

**Authors:** Kelly Garton, Sarah Gerritsen, Fiona Sing, Karen Lin, Sally Mackay

**Affiliations:** School of Population Health, Faculty of Medical and Health Sciences, The University of Auckland, Building 507, Floor 1, 22-30 Park Avenue, Grafton, Auckland, 1023 New Zealand

**Keywords:** Food policy, Digital marketing, Children, Unhealthy food and beverage, New Zealand

## Abstract

**Background:**

Children’s exposure to unhealthy food and beverage marketing has a direct impact on their dietary preference for, and consumption of, unhealthy food and drinks. Most children spend time online, yet marketing restrictions for this medium have had slow uptake globally. A voluntary Children’s and Young People’s Advertising (CYPA) Code was implemented in Aotearoa, New Zealand (NZ) in 2017. This study explores the Code’s limitations in protecting children from harmful food and beverage marketing practices on digital platforms accessible to children.

**Methods:**

A cross-sectional content analysis of company websites (*n* = 64), Facebook pages (*n* = 32), and YouTube channels (*n* = 15) of the most popular food and beverage brands was conducted between 2019 and 2021 in NZ. Brands were selected based on market share, web traffic analysis and consumer engagement (Facebook page ‘Likes’ and YouTube page views). Analysis focused on volume and type of food posts/videos, level of consumer interaction, nutritional quality of foods pictured (based on two different nutrient profile models), and use of specific persuasive marketing techniques.

**Results:**

Eighty-one percent of websites (*n* = 52) featured marketing of unhealthy food and beverages. Thirty-five percent of websites featuring unhealthy food and beverages used promotional strategies positioning their products as ‘for kids’; a further 13% used ‘family-oriented’ messaging. Several websites featuring unhealthy products also had designated sections for children, ‘advergaming,’ or direct messaging to children. Eighty-five percent of all food and drink company Facebook posts and YouTube videos were classified as unhealthy. Twenty-eight percent of Facebook posts for unhealthy products featured persuasive promotional strategies, and 39% premium offers. Nearly 30% of YouTube videos for unhealthy food and beverages featured promotional strategies, and 13% premium offers. Ten percent of Facebook posts and 13% of YouTube videos of unhealthy food and beverages used marketing techniques specifically targeting children and young people.

**Conclusions:**

The voluntary CYPA Code has been in effect since 2017, but the inherent limitations and loopholes in the Code mean companies continue to market unhealthy food and beverages in ways that appeal to children even if they have committed to the Code. Comprehensive and mandatory regulation would help protect children from exposure to harmful marketing.

**Supplementary Information:**

The online version contains supplementary material available at 10.1186/s12889-022-14790-6.

## Background

Unhealthy diets and excess energy intake are modifiable risk factors that, combined, are the greatest contributors to disease and disability in Aotearoa New Zealand (NZ) [1]. Dietary risk factors (i.e. diets low in whole grains, fruit, vegetables, nuts and seeds, and high in sodium, processed red meat, saturated and trans fats, and sugars) are associated with non-communicable diseases (NCDs) such as diabetes, heart disease, dental caries, obesity, certain types of cancer, and poor mental health [2]. Dietary behaviours developed in childhood are important determinants of health throughout the lifecourse [3, 4]. Markers for children’s dietary health in NZ are alarming. NZ has the second highest prevalence of children aged 5–19 with overweight or obesity (39%) in the Organisation for Economic Cooperation and Development, after the United States [5]. In addition, 11.4% of children aged under 15 years have had a tooth extracted due to decay, abscess, or infection [6]. Though data is lacking about the diets of New Zealanders, indicators of child nutrition have consistently shown high fizzy drinks and ultra-processed foods and low fruit and vegetable intake [7–10].

One of the main factors contributing to poor diets and their adverse health outcomes is an unhealthy food environment [11], defined as the collective physical, economic, policy and sociocultural surroundings, opportunities and conditions that influence people’s food and beverage choices and nutritional status [12–14]. Children’s exposure to unhealthy food and beverage (UFB) marketing (i.e. the food marketing environment) has a direct impact on their dietary preference for, and consumption of, these products [15–19]. Moreover, children are particularly vulnerable to the persuasive power of marketing messages and techniques (such as promotional characters, television and movie tie-ins, celebrity and athlete endorsements, in-store marketing and toy co-branding) [15, 19–21]. As efforts to improve children’s diets and reduce the prevalence of childhood obesity in NZ have fallen short, the improvement of food environments through restricting children’s exposure to, and the persuasive power of, UFB marketing practices stands out as a priority in public health nutrition policy [22]. Protecting children from exposure to harmful marketing is also increasingly recognised as a matter of children’s rights, placing the obligation on governments to regulate the food marketing environment [5, 23–27].

Currently in NZ, advertising is self-regulated by the industry-led Advertising Standards Authority (ASA). The ASA introduced the Children and Young People’s Advertising Code (CYPA Code) in October 2017, including rules specific to food and beverage marketing (Table 1). The CYPA Code states that brands and companies cannot target any ‘occasional’ (i.e. unhealthy, as defined by a Ministry of Health Food and Beverage Classification System) food and beverage advertisements to children aged less than 14 years old, and applies a ‘special duty of care’ (undefined) to young people 14–18 years old [28]. However, research has consistently shown that self-regulation (i.e. industry-initiated and voluntary approaches) does not significantly reduce children’s exposure to unhealthy food and beverage marketing [29–32], and a critical review of the CYPA Code by 77 New Zealand health professors expressed concern about the likely lack of impact of this Code [33]. An analysis of complaints made to the ASA about alleged breaches of the Code and the decisions of the Complaints Board and Appeals Board in 2017–2019 found that the majority of complaints were not upheld because the Code was too vague and therefore its interpretation by the Complaints Board was subjective and prone to a narrow application of the Code. The analysis concluded that the ASA system does not adequately protect children from the exposure to, and power of, UFB marketing [34].

**Table 1:** ASA children and young people’s advertising code: key rules specific to food and beverage advertising

ASA CYPA Code: key rules specific to food and beverage advertising[25]	
Principle 1: Social responsibility	
• Rule 1(i) Targeting children: *“Advertisements (including sponsorship advertisements) for occasional food or beverage products must not target children or be placed in any media where children are likely to be a significant proportion of the expected average audience* “(e.g. where 25% or more of the expected audience will be children, in child viewing time zones, content with significant appeal to children, or in locations where children gather).	
• Rule 1(j) Targeting young people: “*A special duty of care must be applied to occasional food and beverage product advertising to young people,”* i.e. “*advertisements must not state or imply that such products are suitable for frequent or daily consumption.”*	
• Rule 1(k) Portion size: “*The quantity of the food in the advertisement should not exceed portions sizes that would be appropriate for consumption on one occasion by a person or persons of the age depicted.”*	
• Rule 1(l) Promotional offers: “*Advertisements featuring a promotional offer of interest to children or young people which is linked to food or beverage products must avoid creating a sense of urgency or encouraging purchase of an excessive quantity for irresponsible consumption.” “There shall be no promotional offers for occasional food and beverage products to children.”*	
Principle 2: Truthful presentation	
• Rule 2(a) Identification: *“It must be clear to children or young people that the advertising is a commercial communication rather than programme content, editorial comment or other non-commercial communication.” “Licensed characters and celebrities popular with children or young people (live or animated) must not obscure the distinction between commercial promotions and programme or editorial content.”*	
• Rule 2 (e) Competitions: *“Where reference is made to a competition, the rules must be clear and the value of prizes and chances of winning must not be exaggerated.”*	
• Rule 2(f) Health benefits: *“Advertisements must not mislead as to the potential physical, social or mental health benefits from consumption of the product.” “Advertisements must not mislead as to the nutritional value of any food or beverage. This includes products high in fat claiming to be low in sugar or sugar free, and products high in sugar claiming to be low fat or fat free.”*	
Principle 3: Sponsorship advertising	
• Rule 3(a) Inclusion of product: *“Sponsorship advertisements must not show an occasional food or beverage product, or such product’s packaging, or depict the consumption of an occasional food or beverage product.”*	
• Rule 3(b): *“Sponsorship advertisements must not imitate or use any parts of product advertisements for occasional food or beverage products from any media.”*	

Recent research on children’s exposure to UFB marketing in media and settings in NZ have demonstrated significant exposure to unhealthy product and brand marketing on television [35, 36], outdoor settings in school zones [37–39], through sports sponsorship [40] and as a ubiquitous presence as they go about their daily life [41]. Children in the latest KidsCam study were exposed to UFB marketing an average of 68 times a day across all settings, which was more than twice their exposure to healthier food marketing (average 26 times per day), and 10% of their total marketing exposure was on screens [42]. Most NZ children have regular access to the internet; 80% of 9–17 year-olds have access to go online when they want or need to, with YouTube, Google, Instagram, Messenger and Facebook the top five most used websites and apps [43]. Vandevijvere et al. (2017, 2018) performed a detailed content analysis of UFB marketing on Facebook, YouTube and company websites between 2014 and 2016. They found that 75% of the most popular food and beverage brand websites included ‘occasional’ foods [44]; 99% of their Facebook posts containing specific products were classified as unhealthy, and 41% of posts used promotional strategies with potential appeal to children; and 77% of YouTube videos containing specific products were classified as unhealthy, while 61% of videos used promotional strategies with potential appeal to children [45].

Exposure to online marketing through Facebook was assessed for a small sample of young people in NZ aged 16–18 years in 2017/2018 in the AdHealth pilot study using a browser extension designed for this purpose, finding that 4% of advertisements users were exposed to were food-related, 98% of which were classified as ‘not permitted to be marketed to children.’ [46] Of these UFB advertisements, 33.7% featured promotional characters and 31.9% premium offers. The mean rate of exposure to UFB advertising was 4.8 ads per hour spent on Facebook [46]. This small feasibility study notwithstanding, in-depth systematic analysis of the marketing children are exposed to on digital platforms while the CYPA Code is in place has not yet been undertaken.

Children spend more and more of their lives online, yet regulation of marketing in digital media has been slow to keep up globally, with most statutory regulations still focused on television advertising. In this study, we sought to describe the extent and nature of UFB advertising/marketing on company-owned digital platforms accessible to children (Facebook, YouTube, and company websites) in NZ after the implementation of the ASA CYPA Code, including how much of this uses promotional techniques which appeal to children, young people and/or families. By doing so, we aimed to explore the limitations of the Code in protecting NZ children from exposure to harmful digital marketing practices.

## Methods

This study involved a cross-sectional assessment of marketing on company websites, Facebook pages, and YouTube channels for the most popular food and beverage brands in NZ. With the objective of exploring the extent and nature of UFB advertising on these platforms, analysis focused on the volume and type of food posts (or videos), level of user interaction with posts (e.g. ‘likes’, shares, comments, views), nutritional quality of foods in posts, and use of specific marketing techniques. We followed a modified protocol for sample selection, data collection and analysis established by the International Network for Food and Obesity/Non-communicable Diseases Research, Monitoring and Action Support (INFORMAS) [12, 47] and applied in previous digital food marketing environment monitoring studies in NZ and internationally [44, 45, 48–50].

### Data collection

#### Company websites

We identified the 64 most popular food and beverage brand websites in NZ by sales and web traffic analysis, using data from Euromonitor (top 50 companies/brands in terms of NZ market share) [51], supplementing with additional websites identified through Alexa (ranking the highest-traffic websites in NZ) [52], and Socialbakers (indicating popularity rank on social media) [53]. All advertising on these websites was documented by screen-shot for content analysis, with data being collected between October 2020 and January 2021.

#### Facebook and YouTube

We monitored social media pages of the largest and most popular food and beverage brands (some representing NZ and Australia in the absence of NZ-specific brand presence) according to NZ market share (Euromonitor), supplementing and triangulating with the number of people who have ‘liked’ the page (Facebook) and number of page views (YouTube channels) as defined by Socialbakers, to end up with a comprehensive sample [53].

Thirty-two food and beverage brands’ Facebook pages were included, and screenshots of their posts recorded over a 6-month period between January–July 2019. This included the 15 most popular packaged food brands and 10 most popular fast-food brands; for beverages there were fewer popular brand pages with a large market share, and we selected the top 7. YouTube channels for 15 food and beverage brands (the top 5 packaged food, 5 fast food, and 5 non-alcoholic beverage) were monitored, with all posted videos recorded between January–December 2019 (a 12-month data collection period required because of the lower volume of advertisements). This study focused on company-posted content and excluded paid-advertising on these platforms. We recorded data separately for fast-food, packaged food and beverage brands as well as for the total sample.

Data collection and coding were conducted primarily by the research assistant (KL) with supervision and training from the principal investigator (SM) who had been involved in the previous NZ assessments. Regular meetings were held to discuss any advertisements difficult to code, and a second research assistant independently coded 25% of the website and 20% of the Facebook and YouTube samples to ensure agreement. Any discrepancies were discussed and resolved, and all coding was reviewed by the first author (KG) during analysis.

### Coding

The coding structure is summarized in Table 2. Advertisements were classified in terms of being for a non-food, non-specific food (e.g. brand-only, product not shown), or specific food product, and in the latter case the featured products were classified as healthy or unhealthy using two different nutrient profiling models: the NZ Ministry of Health Food and Beverage Classification System from 2016 (MOH 2016) classifying foods and beverages as ‘everyday’, ‘sometimes’, or ‘occasional,’ [54] and the nutrient profile system developed by the World Health Organization Regional Office for Europe (WHO-EU) distinguishing between foods permitted or not permitted to be marketed to children [55]. Both models were used because one is domestically developed and applied in the CYPA Code to determine if a food or beverage should be marketed to children (i.e. non ‘occasional’ categories), but the WHO-EU was considered the gold-standard at the time of analysis. We applied the nutrient profiling models to nutrition data collected from company websites, or from an online supermarket. If an advertisement featured multiple products, all were classified but if one product was ‘occasional’ or ‘not permitted’ then the advertisement was categorised as such.

**Table 2:** Summary of coding structure for key indicators

Company advertising characteristics	Coded indicators
Volume of advertisements, consumer engagement and reach	Number of posts per company page/channel (Facebook & YouTube) Number of Likes, Shares, Comments per post (Facebook) Number of Views per post (videos only) Number of Likes, Dislikes, Comments per video (YouTube)
Healthiness of products shown	Specific product(s) shown (Yes/No); Classification according to MOH 2016 [54] • Everyday • Sometimes • Occasional Classification according to WHO-EU [55] • Permitted to be marketed to children • Not permitted to be marketed to children
Protection for children (websites)	Legal information available to parents (embedded in privacy statements companies were legally required to have on their websites, e.g. regarding children’s online privacy); Use of cookies statement (and option to acknowledge/accept or reject cookies that may potentially track children’s online behaviours); Information to parents (e.g. on product safety for children); Age blocks (restrictions) for accessing certain websites; Requirements for parents’ consent(e.g. for membership or registration in competitions when companies interact with children online); Time restrictions (i.e. automatic limits on the amount of time users can spend on a website as a way to reduce marketing exposure).
**Persuasive marketing techniques**	
Promotional strategies	Cartoon/company-owned character; Licensed character; Amateur sportsperson; Celebrity (non-sports); Movie tie-in; Famous sportsperson/team; Non-sports/historical events/festivals (e.g. Christmas, Mother’s Day, World ___ Day); ‘For kids’ (e.g. image of a child, ‘great for school lunches’); Awards won; Sports event; Advercation (e.g. historical facts, general nutrition, sports information, details on product ingredients, other information); Family-oriented messaging to parents that include children’s activities; Sustainable practices (e.g. less plastic, compostable packaging)
Premium offers	Giveaways, Game and app downloads, Gift or collectible; Competitions, Contests, Draws; Deals e.g. “3 for the price of 2”, “20% extra”; Limited edition items; Social charity/fundraising; Limited time product discount; Other
Activity/prompts to promote product	Game; Cooking-related (e.g. demonstrations, recipes); Polls/voting; Commenting, tagging, presence of a hashtag, invitations to like / share; Links to external content; Promo codes; Other
Brand benefit claims	Sensory-based (e.g. taste, texture, appearance, aroma); New brand development; Suggested use (e.g. great for lunchboxes); Suggested users are children or the whole family; Emotive claims (e.g. fun, feelings, popularity, including use of emojis); Puffery (i.e. claiming to be advantageous over other food products or brands); Convenience; Price
Health claims	Health-related ingredients claims; Nutrient content claims (e.g. low fat); Nutrient comparative claims (e.g. reduced fat); General health claims (e.g. healthy diet); Nutrient & other function claim (e.g. calcium good for bones); Reduction of disease risk claims (e.g. Heart Foundation tick); Other claims (e.g. organic)
Other (websites)	Designated children’s section, Advergaming, General gaming

Using a coding structure from previous food marketing environment studies [44, 45, 48], we coded persuasive techniques (‘promotional strategies’, premium offers and claims), with particular attention to those which appeal to children and young people. Promotional strategies considered appealing to children were coded subjectively but included for example cartoons and promotional characters, famous athletes and non-sports celebrities, cross-selling and tie-ins, direct messaging to children, but also the theme of taste and the emotional appeal of ‘fun’ [15, 56, 57] and the portrayal of children or young people. New codes were created for promotional strategies targeting families, and those appealing to sustainability values to explore potential greenwashing of UFB—in particular as sustainability claims are thought to have appeal among parents [58]. Examples are given in Table 2.

### Analysis

We performed basic descriptive statistics on the data collected, and extracted key illustrative examples of the existing UFB marketing on each platform with potential appeal to children or young people.

## Results

The list of food and beverage brands included in the analysis is provided in Supplementary Table 1. A detailed inventory of marketing techniques observed across the three digital platforms (websites, Facebook and Youtube) is provided in Supplementary Table 2.

### Company websites

**Fig. 1:**
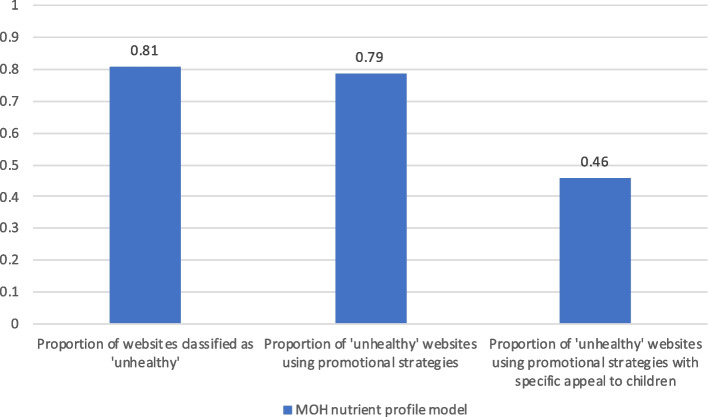
Proportion of food and beverage brand websites classified as ‘unhealthy’, and proportion of such websites using promotional strategies, including those with specific appeal to children

The sample included 49 packaged food brand websites (from 28 parent companies), 10 food retail (fast food) brand websites (from 8 food retail companies), and 5 beverage brand websites (from 2 companies). Of the 64 company websites surveyed, 52 (81.3%) featured marketing of ‘occasional’ products, i.e. only 12 websites (18.8%) had *no* ‘occasional’ products featured (Fig. 1).

#### Persuasive power

Roughly three-quarters of the sampled websites used promotional strategies (78% of the total sample of food and beverage websites, and 79% of websites with ‘occasional’ products).

**Fig. 2:**
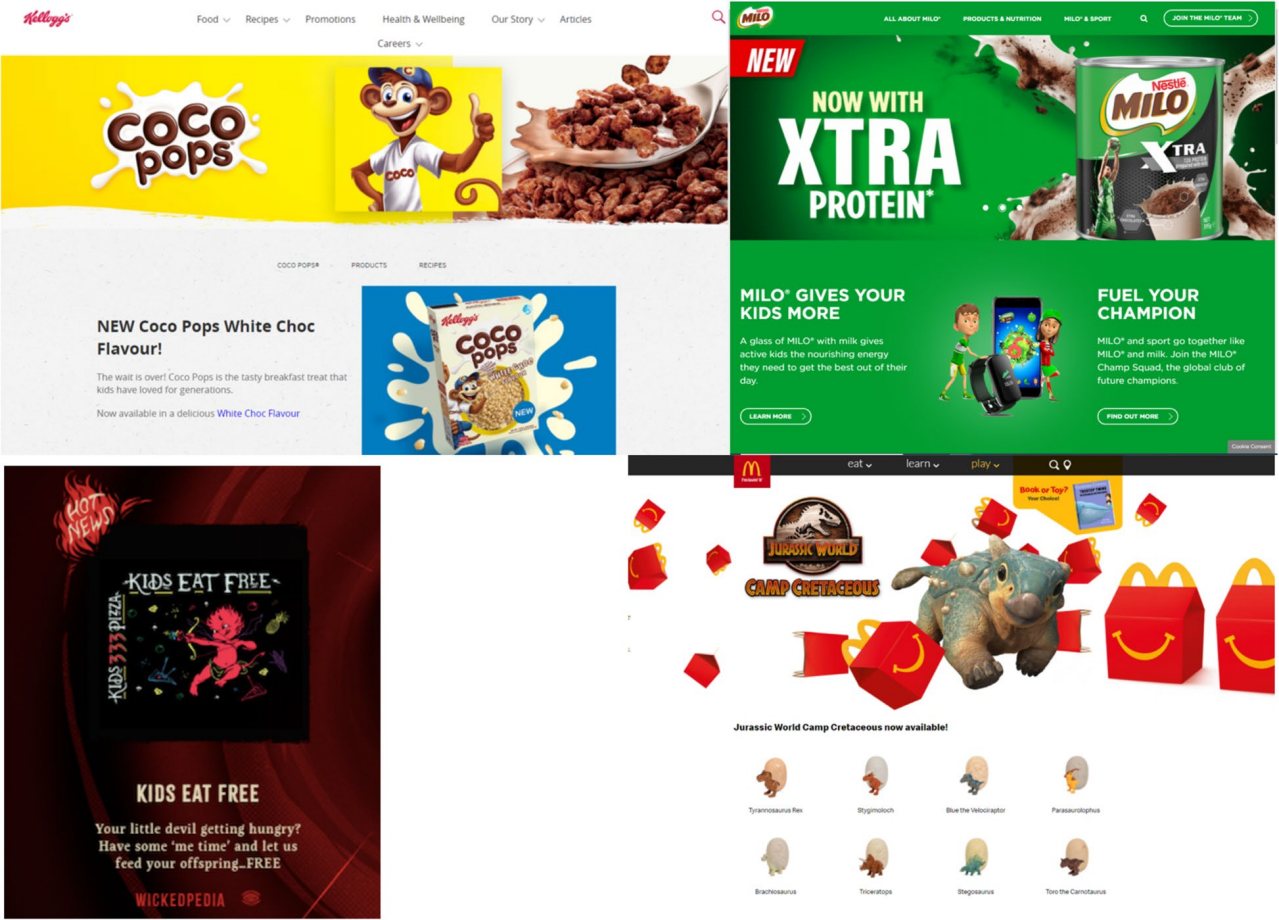
Examples of New Zealand unhealthy food and beverage brand websites. Top left: Kellogg’s Coco Pops featuring branded cartoon monkey and “*the tasty breakfast treat that kids have loved for generations*”; Nestlé NZ’s MILO with ‘Xtra protein’ “*gives active kids the nourishing energy they need to get the best out of their day*”, “*Join the MILO Champ Squad*” app, and professional athlete on package; Bottom left: Hell Pizza “little devils” “kids eat free” promotion; Bottom right: McDonald’s NZ featuring licensed movie tie-in dinosaur toys with ‘Happy Meal’

Among the websites featuring unhealthy (‘occasional’) products, marketing strategies appealing to children were common (examples in Fig. 2). A total of 18/52 (35%) websites featuring ‘occasional’ food and drinks used promotional strategies to position their product(s) as “for kids,” and a further seven (13%) used “family-oriented” messaging. Ten of these websites (19%) featured company-owned cartoon characters or licensed characters that were potentially appealing to children, and seven (13%) featured amateur or famous sports-people or teams.

In addition, 5/52 of these websites for ‘occasional’ products (10%) had designated sections for children (e.g. Kids Zones). Two of the 52 websites featuring ‘occasional’ products (4%) included ‘advergaming’ (a method of interactive marketing in which free downloadable games—often involving brand characters and/or logos—appear on websites to advertise a company or product). One website featuring ‘occasional’ foods included direct messaging to children (to come “find us in the supermarket”) implying a game of hike-and-seek.

Claims about the brand’s benefits were present on nearly every website (98.4%), including sensory-based characteristics (93.8%), emotive claims (76.6%), suggested uses (59.4%), and convenience (43.8%). Notably, 40.4% of the 52 websites featuring UFB (*n* = 21) claimed that the product users were children and/or the whole family.

Three-quarters of the food and beverage websites (75%) featured nutrient content claims and health claims. These were focused on nutrient content (43.8%), health-related ingredient claims (34.4%), general health claims (31.2%), and nutrient comparative claims (26.6%). Of the 52 websites including ‘occasional’ products, 38 (73%) had some form of nutrient content or health claim:

- 20 (38.4%) on nutrient content e.g. “Our [oil] blend includes canola and sunflower oils. It’s cholesterol free (like all vegetable oils) and high in monounsaturated fat.”
- 11 (21.2%) were nutrient-comparative e.g. *“50% less added sugar, 30% less salt*”
- 16 (30.8%) were for health-related ingredients e.g. “A source of prebiotic fibre to nourish your gut bacteria”
- 15 (28.8%) were general health claims e.g. “ … adds 70% more calcium to a glass of milk. Calcium is essential for the growth and development of children as it helps to maintain strong teeth and bones and is important for muscle function, as part of a healthy varied diet.”
- 10 (19.2%) were related to nutrients and other functions e.g. “best sources of fibre, clinically proven to support regularity and promote digestive health”
- 3 (5.8%) were high-level health claims related to reduction of disease, e.g. “provides 2g plant sterols, clinically proven to actively lower LDL cholesterol by up to 9% within 4 weeks”.

Thirty (57.7%) websites overall also featured a range of other claims e.g. gluten-free, organic/all-natural, plant-based, etc.

The company strategies of appealing to consumers’ sustainability values were assessed, identifying that 27 (42%) of all websites promoted their ‘sustainable practices’, including 22 of the 52 UFB websites (42%). This was generally in the form of references to sustainability in company information pages ranging from vague to specific, e.g. sustainable sourcing of cacao for chocolate, ‘Rainforest Certified’ coffee beans, switching to renewable energy in factories, sustainable packaging, carbon-neutral fast-food delivery (e.g. e-bikes), and conservation projects.

#### Protection for children

Fifty-eight (90%) of the food and beverage company websites contained some form of protection for children. The majority of this was in the form of legal information available to parents embedded in privacy statements (e.g. describing adherence to children’s privacy laws)(89% of websites), and/or use of cookies statements allowing the user to accept or reject cookies that may potentially track children’s online behaviour (86% of websites). Information to parents was rare (22%), and other protections were virtually non-existent.

### Company Facebook pages

#### Mean frequency of posts, level of user interaction

Of the 32 food and beverage company Facebook pages assessed, a total of 285 posts were identified, with an average of 9 posts per page, and 1.5 posts per page per month during the recorded period, ranging from 1 (Coca Cola NZ) brand page post (0.17 per month) to a maximum of 43 posts (Domino’s Pizza NZ, 7.17 per month). There was wide variation in consumer interaction with each post in the sample (see Table 3 for measures of spread), with an average of 285 Likes per post (ranging from 0 to 6900), 24 Shares per post (ranging from 0 to 807), and 397 Comments per post (ranging from 0 to 21,000). More than two-thirds (68%) of all posts contained an activity prompt for consumers (e.g. a question to answer in comments, or asking followers to like, comment, tag friends and share their posts), ensuring that their product was seen not only by their followers but also by the followers’ Facebook ‘friends’.

**Table 3:** Volume of advertisements, user engagement, product healthiness and marketing techniques on company Facebook pages for packaged food, fast food, and beverage brands

	Packaged food brands (***n*** = 15)	Fast food brands (***n*** = 10)	Beverage brands (***n*** = 7)	Total 2019 (***N*** = 32 brands)
**Volume and type of posts**				
Total number of posts on all pages, n	102	130	53	285
Number of posts per page: median;	4	10.5	9	7
mean	7	13	7.6	9
(range)	(2 – 18)	(2 – 43)	(1 – 13)	(2 – 43)
Number of posts per month per page: median;	0.67	1.8	1.5	1.2
mean	1.1	2.2	1.3	1.5
**Level of user**^a^ **interaction with posts**				
Likes per post: median;	179	57	70	77
mean	374	171	394	285
standard deviation	± 697	± 297	± 1046	± 655
(range)	(2–5700)	(1–2100)	(0–6900)	(0–6900)
Shares per post: median;	11	3	3	4
mean	47	8	21	24
standard deviation	± 119	± 15	± 59	± 78
(range)	(0–807)	(0–129)	(0–392)	(0–807)
Comments per post: median;	24	42	13	34
mean	551	260	439	397
standard deviation	± 2410	± 770	± 1432	± 1658
(range)	(0–21,000)	(0–6100)	(0–7300)	(0–21,000)
**Healthiness of food and/or beverage products in posts**				
Posts containing a food and/or beverage product, n (%)	95 (93%)	108 (83%)	50 (94%)	253 (89%)
Food and/or beverage products classified as occasional^b^, n (%)	74 (78%)	97 (90%)	49 (98%)	220 (87%)
Food and/or beverage products classified as not permitted to be marketed to children^c^, n (%)	76 (80%)	96 (89%)	48 (96%)	220 (87%)
**Use of marketing techniques**^d^ **in posts**				
Posts with an activity for consumers, n (%)	77 (75%)	78 (60%)	38 (72%)	193 (68%)
Posts with a promotional strategy^d^, n (%)				
• Total	34 (33%)	21 (16%)	23 (43%)	78 (27%)
• Occasional^b^	21 (28%)	14 (14%)	21 (43%)	56 (25%)
• Not permitted to be marketed to children^c^	26 (34%)	14 (15%)	21 (44%)	61 (28%)
Posts with a premium offer^d^, n (%)				
• Total	24 (24%)	65 (50%)	20 (38%)	110 (39%)
• Occasional^b^	15 (20%)	49 (51%)	19 (39%)	84 (38%)
• Not permitted to be marketed to children^c^	17 (22%)	50 (52%)	19 (40%)	86 (39%)

^a^Note, these may include users outside of NZ

^b^Using MOH 2016 Nutrient profile model

^c^Using WHO-Europe nutrient profile model

^d^As defined in Table 2

#### Nutritional quality

**Fig. 3:**
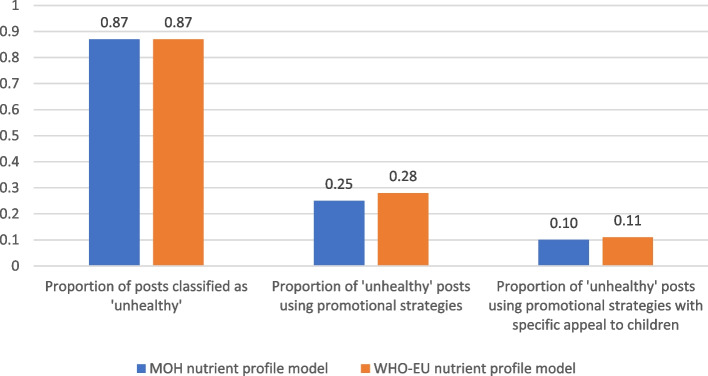
Proportion of food and beverage brand Facebook posts classified as ‘unhealthy’, and proportion of such posts using promotional strategies, including those with particular appeal to children

Eighty-nine percent (*n* = 253) of posts showed one or more specific products that could be assessed against the two nutrient profile models. A large proportion (87%) of the posts that included specific food and beverage products were classed as ‘occasional’ under MOH 2016 or ‘not permitted to be marketed to children’ under the WHO-Europe nutrient profile model (Table 3, Fig. 3).

#### Persuasive power

More than one-quarter of all posts (27%) contained at least one of the coded promotional strategies to attract the consumer’s attention, increase their brand loyalty and make them more appealing to consumers (Suppl Table 2). Frequently used promotional strategies directly positioned the product as relevant to children (e.g. containing images of children, or labelled ‘great for school lunches’) (7.0%, *n* = 20) and featured or tied into non-sports/historical/cultural events or festivals (e.g. Christmas, Anzac Day, Mother’s Day) (6.7%, *n* = 19). Also common were the use of famous sportspersons and teams (e.g. the All Blacks) (6.0%, *n* = 17), followed closely by family-oriented messaging to parents that include children (e.g. family-night recipes, children’s recipes for school term breaks, showing children interacting with parents or siblings) (4.9%, *n* = 14). We recorded only 3 posts promoting sustainability, for example featuring a bottle cap recycling competition, or offering a prize pack with reusable cup.

Premium offers were common (39% of all posts), with popular deals including contests and competitions (16.7%, *n* = 46), limited-time product discounts (9.8%, *n* = 28), and limited-edition items (6.7%, *n* = 19).

**Fig. 4:**
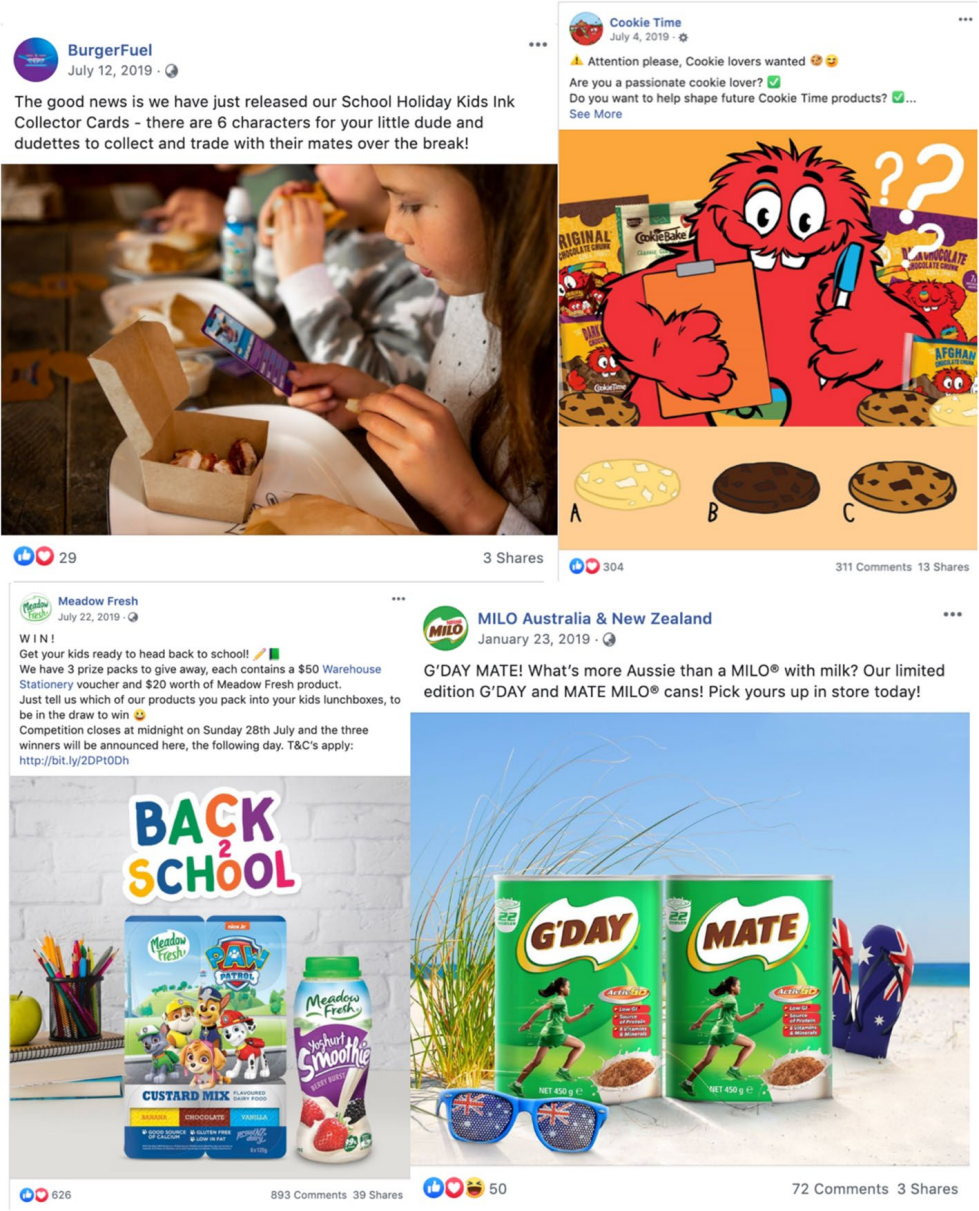
Examples of unhealthy food and beverage marketing targeted to children on New Zealand brand Facebook pages. Top left: BurgerFuel advertising school holiday children’s meals including collector cards; Top right: Cookie Time featuring a contest to become a new cookie tester; Bottom left: Meadow Fresh NZ’s flavoured custard mix ‘back to school’ themed prize pack giveaway of stationery and product voucher, with licensed characters on package; Bottom right: Summer holiday-themed ‘mate’ MILO with child athlete on the package

Of the 87% of posts for UFB, approximately one-quarter used promotional strategies (25% (*n* = 56) of all ‘occasional’ food posts, 28% (*n* = 61) of all ‘not permitted to be marketed to children’ food posts). Approximately 10% of unhealthy food posts (10% (*n* = 21) of all ‘occasional’ food posts, and 11% (*n* = 24) of all ‘not permitted’ food posts) contained promotional elements specifically targeted to children, young people and/or families (see Fig. 3, and examples in Fig. 4).

Thirty-eight percent (*n* = 84) of all ‘occasional’ food posts, and 39% (*n* = 86) of all ‘not permitted to be marketed to children’ food posts, included premium offers. Four of these arguably targeted children directly (e.g. enter your team to win a contest, free school supplies, temporary tattoos or animal-shaped cookie cutter giveaways with product purchase).

#### Brand marketing

**Fig. 5:**
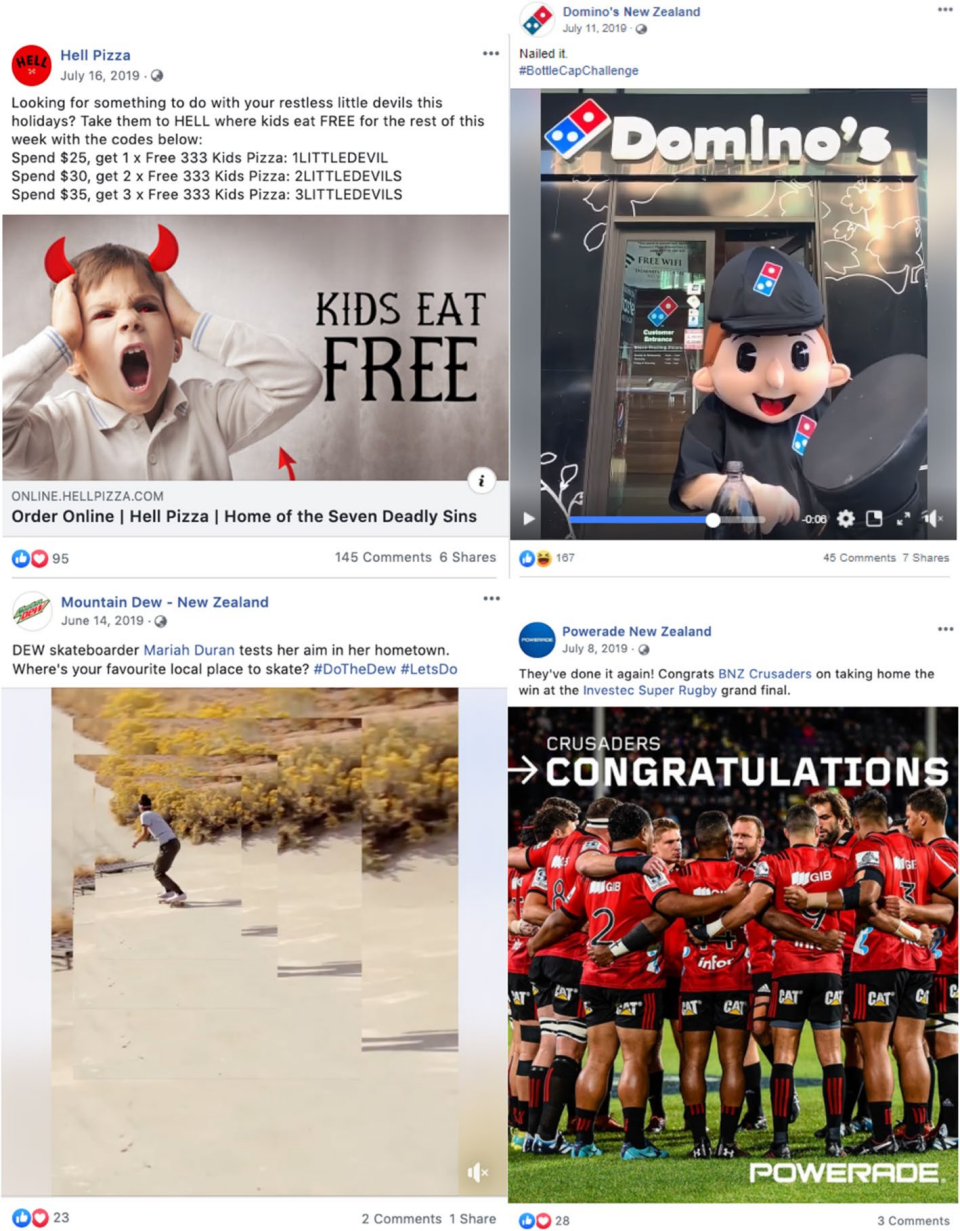
Examples of unhealthy food and beverage brand marketing that do not show specific products on Facebook. Top left: “Kids eat free” premium offers by Hell Pizza; Top right: short video clip of the Domino’s Pizza Man karate-kicking off a bottle cap, tying into a viral video challenge; Bottom left: Mountain Dew NZ video clip of professional skateboarder with #DoTheDew hashtag; Bottom right: Powerade NZ branding of Crusaders rugby team

While most posts (89% overall) promoted specific products, there were also several examples of brand-only advertising that associated the company or brand with children and families, sport, or ‘fun’. This strategy was most common among fast food and beverage brands for which many or most of their products on offer would be classified as ‘occasional’ or ‘not permitted to be marketed to children’ (Fig. 5).

### Company YouTube channels

#### Level of advertising and user engagement

Of the 15 sampled YouTube channels, only 11 brands posted videos during the study time period. A total of 72 videos on all of the channels from 2019 were recorded, with an average of 4.8 videos per channel in the study period ranging from 0 (Sprite Australia-NZ, Streets Ice Cream, Cadbury NZ, Nando’s NZ) to 13 (Kit Kat Australia-NZ and McDonald’s NZ, 1.1 per month). In terms of audience reach, despite a large variation in the sample, we documented an average of 239,757 views per posted video, ranging from 93 to 5,119,353 (see Table 4 for measures of spread). Three videos (two by Kit Kat Australia-New Zealand, one by NESCAFÉ Australia-New Zealand) had over one million views; however, because these brands also represented Australia, the number of views likely include a large proportion of Australian viewers.

**Table 4:** Volume of advertisements, user engagement, product healthiness and marketing techniques on company YouTube channels for packaged food, fast food, and beverage brands

	Packaged food brands (***n*** = 5)	Fast food brands (***n*** = 5)	Beverage brands (***n*** = 5)	Total
**Quantity and views**^a^ **of videos**				
Total number of videos on all channels, n	30	22	20	72
Number of videos per channel: median	6	1	4	4.4
mean	6	4.4	4	4.8
(range)	(0–13)	(0–13)	(0–9)	(0–13)
Number of videos per month per channel: median;	0.5	0.1	0.3	0.4
mean	0.5	0.4	0.3	0.4
number of views^a^ per video: median;	6335	7976	61,006	8909
mean	339,432	66,358	280,985	239,757
standard deviation	± 995,833	± 86,115	± 459,994	± 691,243
(range)	(93–5,119,353)	(385–254,118)	(1256–1,870,052)	(93–5,119,353)
**Nutritional quality of food and/or beverage products in posts**				
Videos containing a food and/or beverage product, n (%)	30 (100%)	8 (36%)	18 (90%)	56 (78%)
Food and/or beverage product videos (n (%)) classified as				
• MOH-Occasional^b^	25 (83%)	8 (100%)	18 (100%)	51 (91%)
• WHO-EU Not permitted to be marketed to children^c^	21 (70%)	8 (100%)	18 (100%)	47 (84%)
**Use of persuasive marketing techniques**^d^ **in posts**				
Videos with an activity for consumers, n (%)	0 (0%)	0 (0%)	0 (0%)	0 (0%)
Number (%) of videos with promotional strategies^d^				
• Total	6 (20%)	16 (73%)	7 (35%)	29 (40%)
• Occasional^b^	4 (16%)	4 (50%)	7 (39%)	15 (29%)
• Not permitted to be marketed to children^c^	2 (9.5%)	4 (50%)	7 (39%)	13 (28%)
Number (%) of videos with premium offers^4^				
• Total	3 (10%)	3 (14%)	0 (0%)	6 (8%)
• Occasional^b^	3 (12%)	3 (38%)	0 (0%)	6 (12%)
• Not permitted to be marketed to children^c^	3 (14%)	3 (38%)	0 (0%)	6 (13%)

^a^Note, these may include viewers outside of NZ

^b^Using MOH 2016 Nutrient profile model

^c^Using WHO-Europe nutrient profile model

^d^As defined in Table 2

#### Nutritional quality

**Fig. 6:**
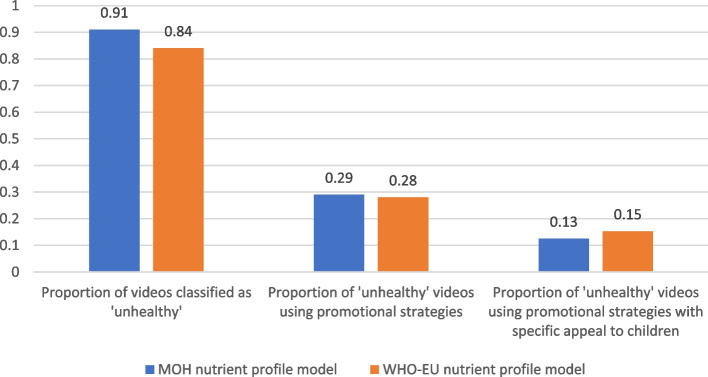
Proportion of food and beverage brand YouTube videos classified as ‘unhealthy’, and proportion of such videos using promotional strategies, including those with particular appeal to children

The nutritional quality of the foods and beverages advertised on YouTube channels was predominantly poor. Of the 11 companies that posted videos in the study period, 9 had posted videos promoting specific products. For 8 of those 9 companies (89%), 100% of their posted videos were for ‘occasional’ or ‘not permitted to be marketed to children’ foods. Overall, 91% of videos featuring products were classed as ‘occasional,’ and 84% classed as ‘not permitted to be marketed to children’ (Fig. 6).

#### Persuasive power of advertising

Forty-two percent of all videos (30/72) used at least one of the coded promotional strategies (Suppl Table 1). The most common was ‘advercation’ with facts about product ingredients (*n* = 16, all of which were from 3 fast-food companies), emphasising ingredient aspects like ‘locally sourced’, ‘all natural’, ‘no additives’, and nutrient content claims like ‘less than 5% sugar.’ Nine videos (12.5%) featured a famous sportsperson or team (*n* = 6), or sports event (*n* = 3). Six videos (8%) included promotional strategies ‘for kids’ and/or ‘for families’, mostly showing children eating with their parents. One video (1.4%) featured a non-sports celebrity (music group). No promotional strategies related to sustainability were observed.

**Fig. 7:**
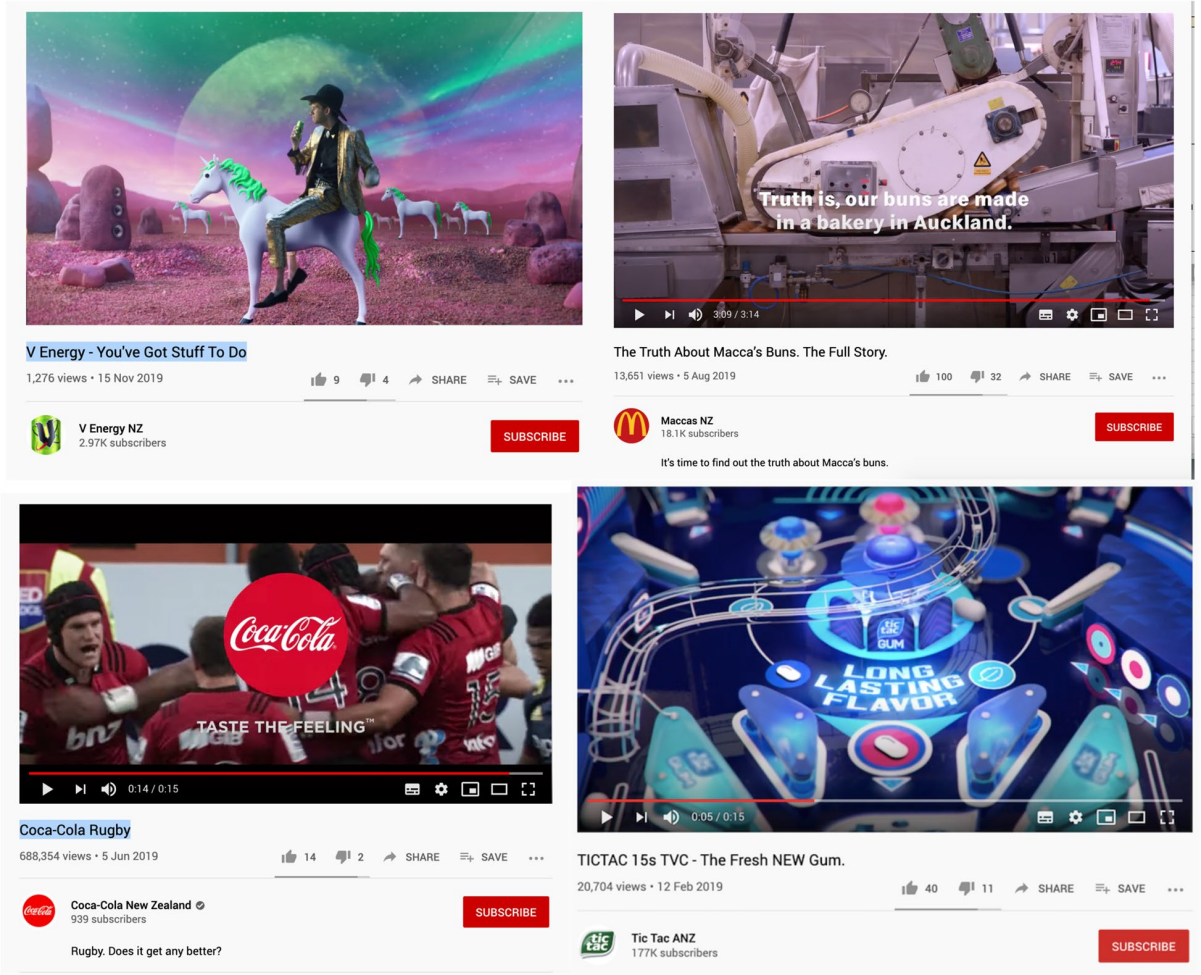
Examples of unhealthy food and beverage marketing on New Zealand brand YouTube channels. Top left: V Energy NZ “*You’ve Got Stuff to Do*” displaying the product; Top right: McDonald’s NZ ‘advercation’ campaign highlighting locally-sourced ingredients, “*Rumour has it our buns are made in a lab … it’s time to find out the truth about Macca’s buns*”; Bottom left: Coca Cola NZ featuring clips of the Crusaders and Highlanders rugby; Bottom right: Tic Tac Australia-NZ video featuring a pin-ball machine game

Of the videos featuring ‘occasional’ (*n* = 51) or ‘not permitted to be marketed’ (*n* = 47) products, approximately 30% used promotional strategies, including portrayals of young people, a popular music group, professional sports teams, and emphasising ‘locally-sourced’ ingredients (Fig. 6, Fig. 7). One example (Fig. 7, 1^st^ image) appeared to appeal to young people with the theme of fantasy, which was not among the coded promotional strategies.

Only 8% of all videos included premium offers, mostly featuring ‘limited time only’ products. All of these videos featuring premium offers were for ‘occasional’ and ‘not permitted to be marketed to children’ products.

#### Brand marketing

Twenty-two percent of all YouTube videos did not feature specific products, so were classified brand-only. However, the majority of brand-only advertising recorded was from fast food chains (representing 64% of their posted videos), and the rest were from beverage brands (10% of their videos). While none of these were deemed to specifically target children, we documented promotional strategies including sport and ‘advercation’ (see Fig. 7).

## Discussion

### Summary of results

This study assessed the extent and nature of UFB marketing by the most popular NZ food and beverage companies on three digital platforms: company websites, Facebook pages, and YouTube channels. Promotional strategies appealing to children were highlighted to assist with monitoring the impact of the CYPA Code introduced in 2017. Overall, there was significant presence of UFB marketing using persuasive techniques. The most prominent strategies included characters (including licensed but more often company-owned); famous athletes, teams, and sports events; tying into historical or cultural events and festivals; ‘for kids’ or family-oriented messaging, including images of children; nutrient and health-related claims; ‘advercation’ with facts about product ingredients; and, on company websites specifically, appeals to consumers’ sustainability values.

More than 80% of the most popular food and beverage company websites featured ‘occasional’ products. Although it is difficult to ascertain children’s actual exposure to UFB marketing through company websites, the extensive use of promotional strategies that specifically appeal to children such as cartoons, characters, ‘for kids’ messaging, and designated children’s sections indicate that some companies are knowingly targeting children through this medium.

On company Facebook pages, 88% of posts contained specific food or beverage products; of these, the majority (86%) were classified as unhealthy. Despite under-13 year-olds technically not being able to register an account on Facebook, the presence of marketing techniques that appeal to children indicates that there is some targeting of children through this medium, particularly when considering all children under the age of 18.

We found a low volume of advertising on food and beverage company YouTube channels. However, most of this (84–91%) was for unhealthy products. There is considerable promotion of UFB products in company videos, using techniques that appeal to children and young people, with the potential for large audience reach.

### Interpretation

This study builds upon previous assessments of the digital food marketing environment in Aotearoa New Zealand; as such, some comparisons can be made, although these are limited due to differences in sampling and coding. Prior to the CYPA (in 2014), of the 70 most popular food and beverage brand websites, 87% featured advercation (compared to 86% in 2020/21), 39% had promotional characters (22% in 2020/21), 19% featured designated children’s sections (vs 11% in 2020/21), advergaming decreased from 13 to 2% in 2020/21, yet general gaming increased from 4 to 8% between the two studies [44]. Premium offers on websites remained relatively constant, recorded on 70% of sampled websites in 2014 and 72% in 2020/21. Seventy-five percent of the websites advertised ‘occasional’ foods in 2014, while 81% of websites featured ‘occasional products in 2020/21. Though direct statistical comparison cannot be made, our results suggest that there is no evidence of significant change in food and beverage company website marketing since the last assessment.

Another assessment carried out in 2015–2016 found a significantly higher frequency of food and beverage advertising on company Facebook pages (average 0.3 post per company per day) and YouTube channels (average 0.8 videos per company per month) than we observed in 2019 [45]. In that study, 64% of Facebook posts contained specific products, and about 99% of these were classified as unhealthy; 41% of posts used promotional strategies with potential appeal to children, 36% used activities/prompts for consumers, and 34% contained premium offers [45]. On YouTube, 84% contained specific products, and 77% of these were unhealthy; 33% of videos had activities for consumers, 61% used promotional strategies with potential appeal to children, and 24% contained premium offers [45]. It is plausible that companies have reduced marketing to children on Facebook and YouTube in response to the voluntary CYPA Code. However, food and beverage companies’ social media presence may have shifted to other platforms, particularly Instagram, Snapchat and Tik Tok which are more popular among children and teens [59] and more difficult to monitor given the use of indirectly paid influencers to market products and viral challenges wherein users generate unofficial brand advertising [60]. The monitoring of a wider range of social media sites and emerging platforms will be important for future research on children’s exposure to marketing.

### Shortcomings of the CYPA code

Despite the existence of the CYPA Code, this research found many UFB advertisements online that appear to target children, young people and families (25–30% of UFB ads on Facebook and YouTube, and on the majority of websites featuring UFB products). Not only is the Code voluntary; it is possible for companies to commit to the Code and keep marketing unhealthy products to children. The CYPA Code narrowly defines ‘child-targeted’ marketing as that which shows specific ‘occasional’ products, uses techniques that only appeal to children (e.g. themes, images, colours, wording, music or language used), and appears in media and settings where children are a large proportion of the audience [28]. The Code does not address the marketing that children actually see and fails to address messaging and media with mixed-age audiences. In addition, the CYPA Code’s exclusion of brand-only advertising introduces a loophole for UFB producers to advertise and build brand loyalty without showing product images.

#### Social media exclusion in the current CYPA

The CYPA Code only addresses advertising in media where children are likely to be a *significant* proportion of the audience (i.e. over 25% of the viewing audience, within children’s programming, in content/media with significant appeal to children, or in locations where children gather) (see Table 1).

Notably, the CYPA Code rule regarding ‘targeting children’ has been interpreted to exclude marketing on social media platforms like Facebook where there is a minimum user age requirement. Sing et al. (2020) found that complaints to the ASA regarding unhealthy food and beverage marketing on Facebook were typically not upheld because children under the age of 13 are not meant to have access to the platform [34].

However, although 13 years is the minimum age for joining Facebook and other social media sites, 26% of mothers of 8-year-olds in NZ recently reported that they do not always follow the age restrictions for social media [61]. Data from the UK and United States indicates that more than half of children aged 12 years and younger are active on social media, and some users are as young as 6 [62, 63]. In NZ, 36% of children (all ages) report using Facebook, though usage is notably higher for those aged 16–17 (75%) [43].

YouTube, on the other hand, has no age controls. YouTube is the most popular website for NZ children aged 5–17 years with over 80% of children accessing the site regularly [43]. Moreover, NZ children’s most common self-reported online activity is watching YouTube videos (75%). Research from the UK found that 48% of children aged 3–4, 71% aged 5–7, and 81% aged 8–11 used YouTube [62].

#### Brand marketing

The CYPA Code discourages the advertising of ‘occasional’ foods targeted at children but does not address the advertising of brands associated with UFB. This aspect of the Code would imply children only respond to product images rather than brands; yet the evidence base shows this is not the case [64]. Brand advertising, which builds brand loyalty, has a powerful influence on children’s food preferences. Our study found significant use of brand marketing by fast food and sugar-sweetened and energy beverage companies on Facebook and YouTube. Mandatory restrictions need to include restrictions on brand marketing as a significant proportion of advertisements (especially fast-food companies’) are brand marketing without promoting specific products.

#### Persuasive advertising targeting parents of young children

The CYPA Code establishes that there should be no promotional offers for ‘occasional’ food and beverage products to children, nor should *licensed* characters and celebrities popular with children or young people blur the distinction between commercial promotions and fact. The Code does not cover marketing designed to persuade parents and caregivers to buy and feed UFB to children. For example: “kids eat free”; family combo meals; children’s health- and nutrition-related claims; or “perfect for kids’ lunchboxes” messages. In the United States (US), where manufacturers face increasing pressure to limit marketing to children, parents have become an increasingly important audience [65]. Parent-directed advertisements for nutritionally poor “children’s foods” often feature nutrition and health messaging and links to an active lifestyle, as well as emotional appeals to family bonding and love [65]. It is therefore critical to understand whether this targeting affects parents’ perceptions and purchases of UFB for their children—currently an underserved area of food marketing research. For instance, parents exposed to certain structure/function claims on toddler milk (formula) in the US have been found more likely to incorrectly believe the product to be as healthy or healthier than cow’s milk, and have greater intentions of giving the product to their child [66].

### Implications for mandatory regulation

Results from this study and others suggest that mandatory regulation (i.e. legislation) is required to ensure compliance and actually reduce children’s exposure to UFB marketing [20, 34, 67–69]. Expert consensus from the World Health Organization (WHO) and academic literature highlights the following characteristics for best-practice: protecting children up to the age of 18, considering all marketing (rather than simply advertising), having a comprehensive definition of child-appealing elements, and restricting not only child-directed content but *all* children’s exposure. We briefly summarise this guidance below.

First, protections must cover children up to the age of 18, in line with the United Nations Convention on the Rights of the Child [5, 23–26]. This would ensure that social media platforms open to young people are not excluded from regulations. Second, the WHO recommends that ‘marketing’ restrictions should cover not only advertising but all commercial communications designed to promote (or have the effect of promoting) increased recognition, appeal, and/or consumption of particular products and services (such as foods high in fats, salt, and sugars) [24, 26]. This would cover the full extent of marketing mediums from billboards to television and online advertisements, product packaging, and promotions at the point-of-sale, including other marketing techniques like product placement, advergaming, sponsorship, and other brand advertisements.

Third, child-appeal can be difficult to define, and there is no international standardized definition of child-appealing marketing elements. For example, a global review of research on marketing to children found a total of 117 techniques highlighted across 133 different studies [70]. Chile’s marketing restrictions, considered to be the ‘gold standard’, define advertising to be targeted to children under 14 *“if it uses, among other elements, children’s characters and figures, animations, cartoons, toys, children’s music, or if it includes the presence of people or animals that attract the interest of children under 14 years old or if it contains statements or fantastic arguments about the product or its effects, children’s voices, language or expressions of children, or situations that represent their daily lives, such as school, playground or children’s games.”* [71] However, it has been argued that definitions of child-appeal should also include design elements like shapes, colours and sound. Mulligan et al. (2021) found that children were drawn to packaging that were perceived as ‘fun’, ‘cool’, ‘exciting’ or ‘interesting’; this included the concept of food or beverages being ‘unconventional’, e.g. in colour, flavour, shape, or name [72]. In addition, the more different things were included on packaging increased the ‘interestingness’ of the product, and therefore its child-appeal [72]. Teens, on the other hand, may be drawn to elements like visual style (e.g. bright or neon colours, fonts, ‘gourmet’ aesthetic, or animated effects), themes (e.g. fashion, sport, sexuality, or technology), special offers, or humour [59]. Regulatory marketing restrictions should therefore include comprehensive and detailed definition of marketing content that has appeal to children of all ages. However, it is increasingly recognised that regulatory focus should be on reducing children’s exposure to UFB marketing, as opposed to simply controlling a set of defined ‘child-directed’ marketing techniques and child-focused media and settings [69, 73, 74]. Therefore, restrictions should apply to any UFB marketing in settings/media to which children are exposed. In line with recommendations from the WHO, the overall policy objective should be ‘of reducing the exposure of children to marketing of unhealthy food and drinks.’ [75].

### Strengths & limitations

This study builds upon assessments carried out from 2014 to 2017 [32, 44, 45], as part of a food environment monitoring initiative aiming to drive accountability for policy change to improve NZ food environments [12]. It provides the most up-to-date snapshot of the digital marketing landscape on these platforms in the country.

One limitation is the subjectivity inherent to coding marketing images, in particular content with appeal to children and young people. We reduced subjectivity by developing detailed coding criteria, definitions and examples in study protocols, with the intention of being conservative in our assessment of harmful marketing (i.e. coding only clear examples). Therefore, we have likely not recorded all of the marketing techniques with appeal to children and young people. Our analysis of nutrient content and health claims on company websites may over-represent the occurrence of such claims being about UFB products, as we collected and coded claims appearing on websites featuring *any* ‘occasional’ products, so these were not necessarily about the ‘occasional’ products specifically.

Digital marketing is also difficult to monitor, as the landscape is rapidly changing, with new platforms constantly emerging, and also because targeted online advertising means exposure is different for each individual. A notable limitation is that this study did not assess food and beverage marketing on all digital platforms, in particular some with emerging popularity among young people, such as Instagram, Snapchat and Tiktok.

On the platforms we did include, this study did not capture *paid* advertising by the major food and beverage companies. It therefore provides only a small picture of the digital marketing to which children, young people and adults may be exposed. However, company website, Facebook and YouTube marketing strategies are indicative of the broader marketing landscape, assuming that any advertising campaigns introduced on a company website or social media page will also likely feature throughout media that were not monitored as part of this study. This study also shows the need for mandatory regulation of marketing to children to include company-owned digital media platforms in addition to paid advertising content.

## Conclusions

The voluntary CYPA Code has been in effect since 2017, but children and young people are likely still exposed to a range of persuasive UFB advertising on digital platforms. New Zealand needs a comprehensive and mandatory regulatory restriction to protect children up to the age of 18 years from UFB marketing. This study, taken in combination with other recent assessments of other NZ food marketing environments and analyses of decisions regarding complaints filed, indicates major gaps in the CYPA Code which provide loopholes for companies to continue marketing UFB products to children. Marketing restrictions in NZ must include social media platforms and media with mixed-age audiences, should apply to brand-only marketing in addition to product names and images, and should also cover marketing that targets parents and families as this encourages children’s consumption of unhealthy products.

## Supplementary Information

**Additional file 1: Supplementary Table 1.** Food and beverage brands included in the analysis. **Supplementary Table 2.** Prevalence of marketing techniques on company websites, Facebook pages and YouTube channels.

## Data Availability

The dataset used and analysed during the current study are available from the corresponding author upon reasonable request.

## Abbreviations

NZ: Aotearoa New Zealand
NCDs: Non-communicable diseases
UFB: Unhealthy food and beverage
ASA: Advertising Standards Authority
CYPA: Children and Young People’s Advertising
INFORMAS: International Network for Food and Obesity/Non-communicable Diseases Research, Monitoring and Action Support
MOH 2016: NZ Ministry of Health Food and Beverage Classification System from 2016
US: United States of America
WHO-EU: World Health Organization Regional Office for Europe
