# Supplementary material for: Unhealthy food and beverage marketing to children on digital platforms in Aotearoa, New Zealand
Source: BMC Public Health. 2022 Dec 22;22:2407. doi: 10.1186/s12889-022-14790-6 (PMC9773428; doi:10.1186/s12889-022-14790-6)
Supplement: Supplementary file 1 — Additional file 1: Supplementary Table 1. Food and beverage brands included in the analysis. Supplementary Table 2. Prevalence of marketing techniques on company websites, Facebook pages and YouTube channels. [file 12889_2022_14790_MOESM1_ESM.docx]

**Supplementary Table 1.** Food and beverage brands included in the analysis

|  | **Included in website analysis (2020/2021)** | **Included in Facebook analysis (2019)** | **Included in YouTube analysis (2019)** |
| --- | --- | --- | --- |
| Packaged food brands | Arnott’s, Campbell’s, Bluebird, Doritos, Cookie Time, Dairyworks, Edmonds Cooking, Share the Fantastic, Farrah’s, Anchor, Mainland, Tip Top Ice Cream, Burgen, Tip Top Bakery, Meadow Fresh, Meadowlea, Puhoi Valley, Eta, Eta Salads, Griffins, Huntley & Palmer, Nice & Natural, Heinz, Wattie’s, Heller’s, Kiwi Bacon, Kellogg’s, Lewis Road Creamery, M&M’s, Masterfoods, Uncle Ben’s, McCain, Philadelphia Australia & NZ, Cadbury, Pascall, My Food Bag, Kit Kat, Maggi, Sanitarium Health Food Co NZ, So Good, Weet-Bix, Sealord, Tasti, Tegel, Chelsea Sugar, Flora, Whittaker’s, Extra | Tip Top Ice Cream, Lewis Road Creamery, Share the Fantastic, Edmonds, Philadelphia Australia & NZ, Kiwi Bacon, Puhoi Valley, Cookie Time, Chelsea Sugar, Tasti, Heinz Wattie’s, Farrah’s, Meadow Fresh, Anchor, Kapiti | Tic Tac Australia & NZ, Kit Kat Australia & NZ, Streets Ice Cream, Cadbury NZ, Sealord |
| Fast-food brands | Domino’s NZ, Hell Pizza, MCDonalds, Pita Pit, Pizza Hut, Burger King, Carl’s Jr, KFC, Subway, Starbucks NZ | Domino’s NZ, Burger King NZ, Subway NZ, Carl’s Jr, Starbucks NZ, Pita Pit NZ, Hell Pizza, Nando’s NZ, Wendy’s, Burger fuel | Hell Pizza, KFC NZ, Maccas NZ (McDonalds), Domino’s NZ, Nando’s NZ |
| Beverage brands | Charlie’s Drinks, Coca Cola NZ, MILO NZ, Powerade NZ, Pepsi (Frucor Suntory), V Energy NZ | Coca Cola NZ, Nestle NZ (MILO), Lemon & Paeroa, V Energy NZ, Mountain Dew NZ, Charlie’s Drinks, Powerade NZ | NESCAFE Australia & NZ, V Energy NZ, Sprite Australia & NZ, Coca Cola NZ, Gatorade NZ |

**Supplementary Table 2**. Prevalence of marketing techniques on company websites, Facebook pages and YouTube channels

|  | **Prevalence (n (%))** | | |
| --- | --- | --- | --- |
| **Marketing techniques** | **Websites**  **(N=64 websites)** | **Facebook (N=285 posts)** | **YouTube**  **(N=72 videos)** |
| Designated children’s section | 7 (10.9%) | n/a | n/a |
| Advergaming  General gaming | 2 (3.1%)  4 (6.3%) | n/a | n/a |
| **Promotional ‘power’ strategies**  Cartoons, company owned  Licensed characters  Famous sports persons/teams  Famous celebrities  Amateur sport persons  Movie / TV tie-ins  Non-sports/historical events/festivals  ‘For kids’  Family-oriented messaging  Awards received  Sports event  Sustainable practices | **50 (78.1%)**  11 (17.2%)  3 (4.7%)  5 (7.8%)  2 (3.1%)  4 (6.3%)  0 (0.0%)  8 (12.5%)  25 (39.1%)  15 (23.4%)  11 (17.2%)  7 (10.9%)  27 (42.2%) | **78 (27.3%)**  11 (3.9%)  0 (0.0%)  17 (6.0%)  0 (0.0%)  3 (1.1%)  0 (0.0%)  19 (6.7%)  20 (7.0%)  14 (4.9%)  2 (<1%)  10 (3.5%)  3 (1.1%) | **13 (18.1%)**  0 (0.0%)  0 (0.0%)  6 (8.3%)  1 (1.4%)  0 (0.0%)  0 (0.0%)  0 (0.0%)  5 (7.0%)  5 (7.0%)  0 (0.0%)  2 (2.8%)  0 (0.0%) |
| **Educational material (‘advercation’)** | **56 (87.5%)** | **8 (2.8%)** | **16 (22.2%)** |
| **Activity prompts** | **n/a** | **193 (67.7%).** | **0 (0%)** |
| **Premium offers**  Giveaways (incl. gift/collectibles, game/app downloads)  Competitions, contests, draws  Fundraising (incl. social charity)  Vouchers (e.g. 3 for 2, 20% off)  Sample offers  Limited edition items  Limited time offers  Other | **46 (71.9%)**  6 (9.4%)  13 (20.3%)  16 (25.0%)  12 (18.8%)  2 (3.1%)  n/a  n/a  18 (28.1%) | **110 (38.6%)**  17 (6.0%)  46 (16.1%)  1 (<1%)  17 (6.0%)  n/a  19 (6.7%)  28 (9.8%)  3 (1.1%) | **6 (8%)**  0 (0.0%)  1 (1.4%)  0 (0.0%)  0 (0.0%)  n/a  5 (6.9%)  0 (0.0%)  0 (0.0%) |
| **Brand benefit Claims**  Sensory-based  Emotive claims  Suggested uses for children / suggested users are children or the whole family  Convenience  Puffery  Price  New brand development | **63 (98.4%)**  60 (93.8%)  49 (76.6%)  38 (59.4%)  28 (43.8%)  9 (14.1%)  11 (17.2%)  n/a | **236 (82.8%)**  55 (19.3%)  118 (41.4%)  81 (28.4%)  27 (9.5%)  2 (<1%)  43 (15.1%)  35 (12.3%) | **51 (70.8%)**  25(34.8%)  4 (5.6%)  17 (23.6%)  5 (6.9%)  4 (5.6%)  0 (0.0%)  10 (13.9%) |
| **Health Claims**  Nutrient content  Health-related ingredients  General health claims  Nutrient comparative claims  Nutrient & other function  Other e.g. organic | **48 (75.0%)**  28 (43.8%)  22 (34.4%)  20 (31.2%)  17 (26.6%)  15 (23.4%)  38 (59.4%) | **27 (9.5%)**  5 (1.8%)  7 (2.5%)  5 (1.8%)  2 (<1%)  2 (<1%)  12 (4.2%) | **25 (34.7%)**  6 (8.3%)  1 (1.4%)  12 (16.7%)  0 (0.0%)  3 (4.2%)  7 (9.7%) |
| **Protection for children**  Legal information available  Use of cookies statement  Information to parents  Age blocks  Parents’ consent  Time restrictions | **58 (90.6%)**  57 (89.1%)  55 (85.9%)  14 (21.9%)  0 (0.0%)  1 (1.6%)  0 (0.0%) | n/a | n/a |
